# Event and Comment

**Published:** 1905-07-15

**Authors:** 


THE
### DENTAL REGISTER.

Vol. LIX. July 15, 1905. No. 7.

### EVENT AND COMMENT.

### Dental Society Work.

There seems to be a decidedly increasing interest in the work of the dental society. We recently attended a meeting of the Northern Ohio Dental Society, in Cleveland, and were quite surprised to learn that over 400 dentists were in attendance, and that nearly fifty exhibitors of various supplies were present. The program was a good one and there were a large number of clinics. It is a little difficult to say just what contributed to such a large meeting. Two of the gentlemen who read papers came all the way from Philadelphia and several visitors were there from Michigan, but the large attendance came from the State of Ohio. It seemed as though every dentist from the northern part of the state must certainly have attended the meeting. The executive committee worked hard to get a large meeting, and did everything possible to provide a program that would be interesting and up-to-date, but similar effort has been made before with no such results. The Illinois State Society has just held its annual meeting with the largest attendance in its history, nearly one thousand. But this resulted, to some extent, from the fact that during the last year the entire state has been systematically canvassed for members of local and the state society, until the membership of this state society has now reached more than twelve hundred.

The Pennsylvania State Society has just sent out a most elaborate and ornately printed program of its coming meeting in the finest hotel in the world at Philadelphia, offering eight or ten papers, more than sixty clinics, and over fifty exhibitors, and expects an attendance of nearly a thousand dentists. The same kind of news comes from every part of the country, and cannot be explained on the statement that the executive committees are unusually active. Is it possible to account for all this interest in society meeting on the basis of such individual efforts as we have indicated?

There is a larger number of dentists in practice now than ever before. The public is being educated to better dentistry, and is demanding a higher order of service than ever before. Competition is stronger than before, and the successful practitioner is the one who is capable of fulfilling the demands of the public. The average practitioner realizes that he cannot get what he needs by reading the dental journals, good as they are, but he has found out that he must see the men at close range do the things he eads about in the journals. And the only way he can accomplish this is by attending the meetings and getting next to these men and becoming inoculated, as it were, with their enthusiasm. This also is necessary that he may see what is being supplied by the manufacturer for facilitating the exacting technical work now demanded from the modern practitioner. It may be a wave of enthusiam that will break and spend itself on a shore of indifference by and by, but let us hope that it will result in a revival of the spirit of inquiry that shall result in more perfect knowledge and skill and professional advancement.

**To Whom the Good?**

The editor of the *Dental Cosmos* in the June issue comments on some statements made by the President of the National Board of Examiners, who arraigned the colleges for not requiring higher educational attainments of students, for admission to the course of study, and for not teaching manual training; he also took occasion to say that the majority of colleges are conducted for the money to be made out of them and that some advertise in an unethical a manner as the dental parlors. The editor of the *Cosmos* makes the point that more than two-thirds of the states do not recoginze a college degree, but compel candidates for license to practice to submit to an exhaustive examination on all branches of the curriculum, and are therefore morally and legally responsible, should an unfit man be permitted to practice. And further, that in many states it is not necessary that a candidate for a license should have matriculated in a college at all before applying for the license; thus enabling men to cram for an examination and pass it, without any adequate training in technical dentistry. The editor also asks the question whether it is probable that a larger percentage of men are practicing dentistry as a benevolence than are managing dental colleges for the good of professional education. He claims that professional ethics is taught in every dental college in the country, both by precept and example. He maintains that the fault is not as much with the methods of the colleges, which may not be perfect, and which are being improved all the time, as it is with the system of education prevailing throughout the country. The colleges cannot demand a product that our schools will not supply. It is a mistaken notion that a training in hand craft and ability to write intelligibly the English language will cure this evil. The mind and the hand must be trained to work sympathetically and coordinately, and this kind of education must be carried into the higher branches of science and art to secure the best preparation. In other words, it is not the amount, but the quality of the preliminary education that will make for an improvement. It seems to us that we spend altogether too much time in a kind of abnormal introspection and accusation of each other for supposed short comings. There is no doubt, perhaps, that we ought to manage our colleges better, and equip our graduates to meet the requirments of the profession better than we do; and our public schools ought to send us students with better preparation. But, as the boy said, "the Lord could'nt make a ten year old calf in a minute," so we can't lift ourselves by our own boot straps. The profession must create a sentiment for higher ideals, the examiners must set us higher standards and the colleges, some a little belated, perhaps, but all in time will either come up to these ideals and standards or go out of business. Our colleges are what we make them, let us either support or abolish them.

**Raising College Fees.**

We are informed that the effort to get the necessary majority of three-fourths of the dental colleges in the National Association of Faculties to agree to raise their annual fees to one hundred and fifty dollars has failed, although more than one-half of the colleges responded favorably. Dental Education has become such an expensive luxury that it seems absolutely necessary that students shall pay more for such training as the legal authorities are now demanding. The technical departments have expanded so much in the last five years that former wholesale methods of imparting instruction have had to be abandoned, and more individual work is now required. Besides this plan which involves more instructors, it is found that dentists who are capable of doing this kind of work will not now do it for the sake of a college connection. It requires so much loss of time from ones practice that he can't afford to do it with out compensation. To pay all these teachers and keep up a modern plant in the way of building, etc., costs much more than under former conditions. Then again the medical science branches are not now taught by the lecture system. But the laboratory plan must be used to get results, and both, men and equipment of this kind, costs lots of money. The prospects seems to be that we shall have fewer colleges or larger fees will have to be paid by future students.

**Dental Journalism.**

A new dental journal is announced to appear in September, from Cleveland. It is announced that it will be conducted on somewhat new lines, and its history will be watched with interest. It will have four editors, each one responsible for one of the following four departments: Operative Dentistry will be conducted by Dr. W. J. Jackman; Prosthetic Dentistry by Dr. George H. Wilson; Orthodontia by Dr. V. H. Barnes, and Humanitarian Dentistry by Dr. W. G. Ebersole; an excellent staff. The intention is to solicit original contributions from the profession and pay the authors for such contributions. The motto of the journal will be to "educate, pacify, purify and comfort." The publishers promise to make it also an artistic production. Articles will be fully illustrated and the cover page will be changed each month. We don't know how the profession will take to such a venture, but we are of the impression that the cost will be out of proportion to the appreciation, if all these promises are fulfilled. But why shouldn't we make an effort to educate ourselves into higher art realms, and why shouldn't we be paid for the knowledge we have worked out with great cost, and which we have freely given to all who are pleased to pay the small sum of one dollar per year for a good readable journal? Success to the new journal.

**Fellowship Medal.**

At the suggestion of Dr. William Jarvie, of New York City, the New York State Society has adopted a plan for recognizing the efforts of men who have or are spending their lives in the interest of the profession. Dr. Jarvie has given to the society one thousand dollars, the interest of which is to be used to buy a gold medal to be presented to such persons as may be thought worthy to be named as Fellows of the New York State Society, only one to be named each year. A committee of five has been appointed by the society and this year the honor goes to Dr. G. V.

Black, of Chicago. This is a very nice thing to do, and the committee certainly made no mistake in its selection of the first man who should receive the honor. This selection may not make Dr. Black any more universally honored than he now is, but it should please him to know that his laborious work is so appreciated as to merit this public recognition, from a great representative society. We congratulate both the New York society and the recipient of the fellowship. This is the kind of hero worship that should be profitable to our profession and bring encouragement to other hard workers.

